# Supplementary material for: Mechanism of the electrochemical hydrogenation of graphene
Source: Nat Commun. 2025 Nov 28;16:10741. doi: 10.1038/s41467-025-65771-3 (PMC12663420; doi:10.1038/s41467-025-65771-3)
Supplement: Supplementary file 1 — Supplementary Information [file 41467_2025_65771_MOESM1_ESM.pdf]

Supplementary Information for

Mechanism of the electrochemical hydrogenation of graphene

Y.-C. Soong[1,2+], H. Li[1+], Y. Fu[1,2+*], J. Tong[1,2], S. Huang[1,3], X. Zhang[1,2], E. Griffin[1,2], E. Hoenig[1,2], M. Alhashmi[1,2], Y. Li[4,5], D. Bahamon[4,5], J. Zhong[6], A. Summerfield[2], R. N. Costa Filho[7], C. Sevik[8], R. Gorbachev[1,2], E. C. Neyts[9], L. F. Vega[5,6], F. M. Peeters[10,8,7], M. Lozada-Hidalgo[1,2*]

[1] Department of Physics and Astronomy, The University of Manchester, Manchester M13 9PL, UK
[2] National Graphene Institute, The University of Manchester, Manchester M13 9PL, UK
[3]Department of Chemical Engineering, Centre for Integrated Materials, Processes & Structures (IMPS), University of Bath BA2 7AY, Bath, UK
[4]Research and Innovation Center on CO2 and Hydrogen (RICH Center) and Chemical and Petroleum Engineering Department, Khalifa University of Science and Technology, PO Box 127788, Abu Dhabi, United Arab Emirates
[5]Research and Innovation Center for graphene and 2D materials (RIC2D), Khalifa University of Science and Technology, PO Box 127788, Abu Dhabi, United Arab Emirates
[6]Department of Chemistry, The University of Manchester, Manchester M13 9PL, UK
[7]Departamento de Física, Universidade Federal do Ceará, 60455-900 Fortaleza, Ceará, Brazil
[8]Departement Fysica, Universiteit Antwerpen, Groenenborgerlaan 171, B-2020 Antwerp, Belgium
[9]Department of Chemistry, University of Antwerp, Universiteitsplein 1, 2610 Antwerp, Belgium
[10]Nanjing University of Information Science and Technology, Nanjing 210044, China.

[+]These authors contributed equally to this work.

[*]Corresponding authors: yangming_fu@outlook.com; marcelo.lozadahidalgo@manchester.ac.uk.

The PDF file includes:

Supplementary Figures

Supplementary Figure 1 | Potential energy profile for graphene hydrogenation as a function of lattice corrugation.

Supplementary Figure 2| Experimental devices.

Supplementary Figure 3 | Calculations of H_2 formation via an Eley-Rideal process.

Supplementary Figure 4 | Calculations of H_2 formation via a Langmuir-Hinshelwood process.

Supplementary Figure 5 | Experimental circuit for time-resolved measurements of the hydrogenation transition.

Supplementary Figure 6 | Conductivity of HTFSI and DTFSI.

Supplementary Figure 7 | NMR characterisation of DTFSI electrolyte.

Supplementary Figure 8 | Raman characterisation of deuteration transition.

Supplementary Figure 9 | Corrugated graphene roughness.

Supplementary Figure 10 | Raman characterisation of hydrogenation transition in corrugated graphene samples.

Supplementary Table 1 | Statistics.

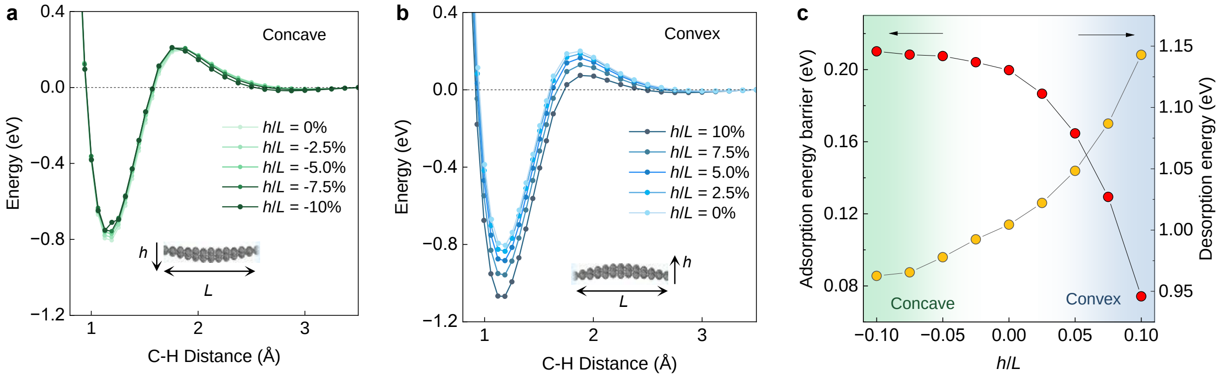

Supplementary Figure 1 | Potential energy profile for graphene hydrogenation as a function of lattice corrugation. DFT calculation of potential energy vs distance for a proton approaching a carbon atom in graphene along a trajectory perpendicular to the basal plane. Panels **a** and **b** show this calculation for different convex and concave structures with height (*h*) to base (*L*) *h*/*L* ratios. Negative (positive) *h*/*L* values indicate concave (convex) structures. Insets, schematics of typical lattice structures. **c** Energy barrier (red data points) and desorption energy (yellow data points) obtained from data in panels **a** and **b**.

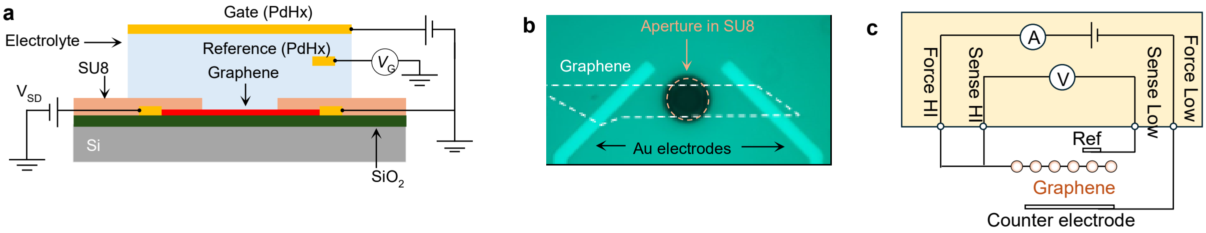

Supplementary Figure 2| Experimental devices. **a** Schematic of experimental devices. **b** Optical image of devices. Dashed white lines mark the graphene flake edges. Dashed orange circle (15 μm diameter), aperture in the SU-8 seal. **c** Measurement circuit for cyclic voltammetry with Keithley sourcemeter.

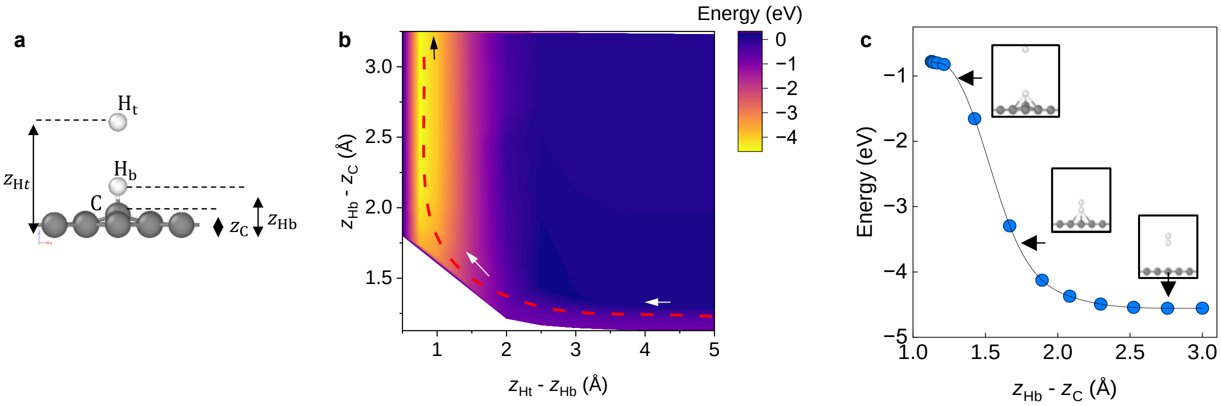

Supplementary Figure 3 | Calculations of H_2 formation via an Eley-Rideal process. a Schematic of model system, in which a proton approaches another proton already adsorbed in graphene. **b** Potential energy surface for the desorption process. *x*-axis, distance between the two protons. *y*-axis, distance between the initially adsorbed proton and the graphene lattice. Dotted red curve, path of minimum energy followed by incoming proton. Arrows mark the direction of decreasing energy along the path. **c** Energy of the system vs distance between adsorbed proton and carbon atom. The energy drops by several eV when the two protons form a H_2 molecule and move away from the graphene lattice. Insets show the system at given points along this trajectory.

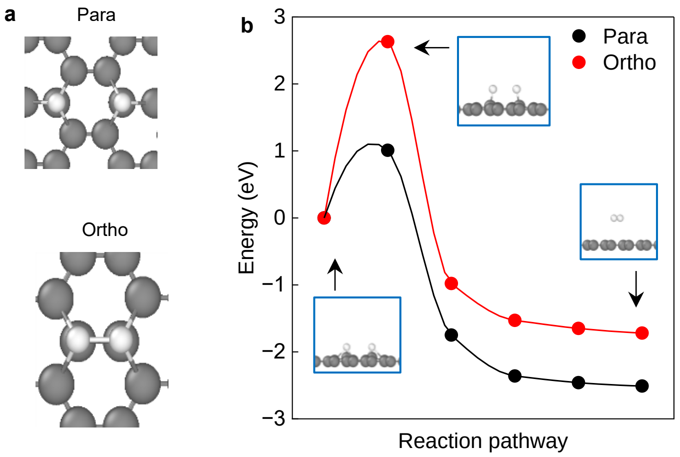

Supplementary Figure 4 | Calculations of H_2 formation via a Langmuir-Hinshelwood process. a Schematic of adsorbed dimer configurations. **b** Energy of the system for different positions of the two protons along the reaction pathway for Para (black) and Ortho (red) proton dimers. Solid lines, guide to the eye. Insets show the system at given points along the reaction pathway.

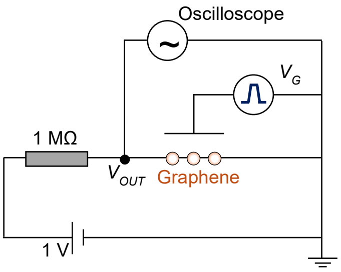

Supplementary Figure 5 | Experimental circuit for time-resolved measurements of the hydrogenation transition. A bias of 1 V is applied between the graphene and a 1 MΩ series resistor. An alternating gate voltage is sourced with a waveform generator, and the output voltage is recorded with an oscilloscope.

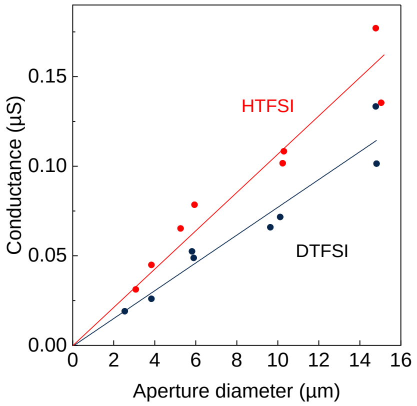

Supplementary Figure 6 | Conductivity of HTFSI and DTFSI. Conductance of devices consisting of a circular hole etched in a silicon nitride substrate; HTFSI/DTFSI electrolyte on both sides; and two PdH_x/PdD_x electrodes. The extracted conductance for HTFSI is ≈35% higher than for DTFSI electrolyte. Solid lines, best linear fit to data.

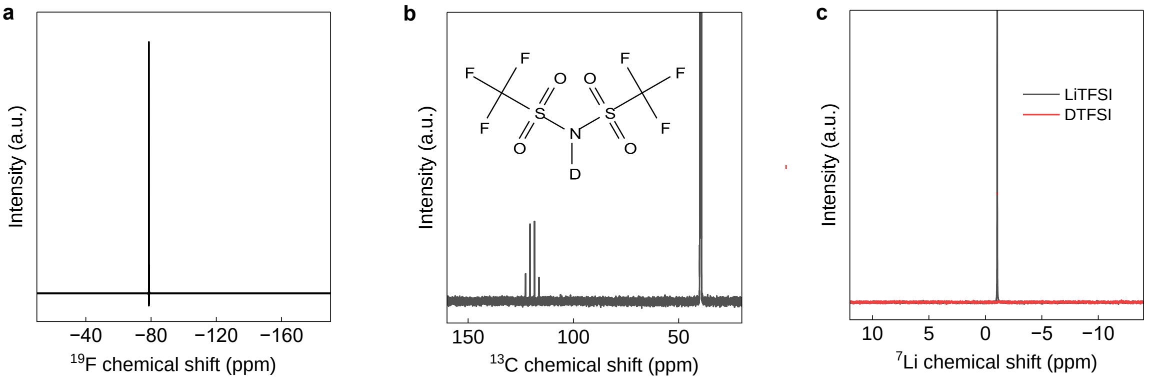

Supplementary Figure 7 | NMR characterisation of DTFSI electrolyte. **a** ^{19}F NMR (500 MHz, DMSO-d_6, 298 K) of DTFSI. The spectrum shows a singlet characteristic fluorine peak at a chemical shift of -78.72 ppm, indicating the existence of $(CF_3SO_2)_2N$-anion[64]. The positions of the three fluorine nuclei in $-CF_3$ are interchangeable by rapid rotation of the carbon-fluorine bonds, so the three fluorine nuclei are chemically equivalent. The two $-CF_3$ groups are symmetric in the $(CF_3SO_2)_2N$-anion, thus the six-fluorine nuclei are chemically equivalent. **b** ^{13}C NMR (151 MHz, DMSO-d_6, 298 K) of DTFSI. The spectrum shows one set of quartet peak of carbon from two symmetric $-CF_3$ groups, at a chemical shift of 119.5 ppm, indicating the existence of $(CF_3SO_2)_2N$-anion[65]. The spin-spin coupling between nuclei of carbon and fluorine causes the spin splitting of carbon, resulting in a quartet peak of carbon. The C-F coupling constant $J_{C\text{-}F}$ (321.6 Hz) is determined by timing the chemical shift difference (2.13 ppm) with Hertz number of the instrument (151 MHz). Inset, model of DTFSI molecule. **c** ^{7}Li NMR (400 MHz, DMSO-d_6, 298 K) of DTFSI and LiTFSI.

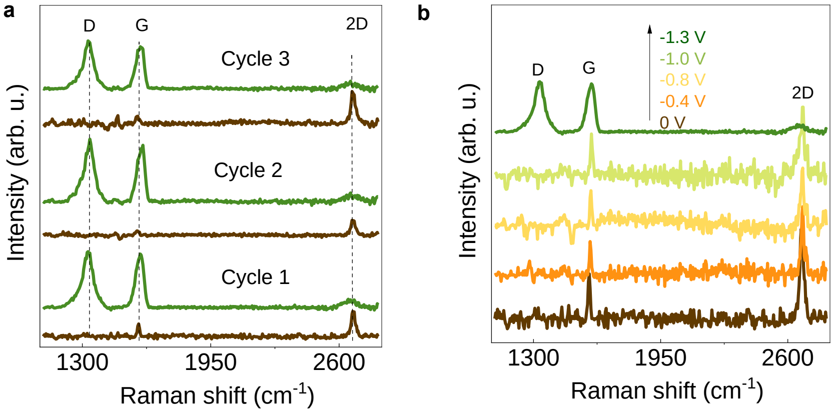

Supplementary Figure 8 | Raman characterisation of deuteration transition. a The Raman spectra of deuterated samples are accompanied by a sharp D band and a smeared 2D band. The samples could be deuterated and de-deuterated multiple times. **b** Raman spectra of samples during a deuteration cycle. The signatures of the deuteration transition appear for a gate voltage of about -1.3 V vs NP.

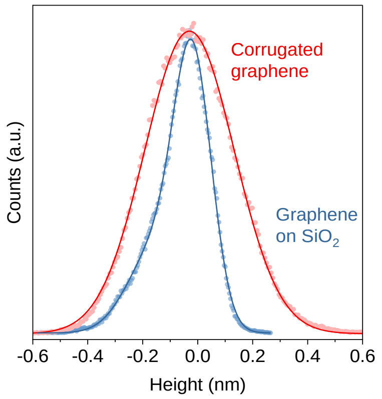

Supplementary Figure 9 | Corrugated graphene roughness. Height distribution for graphene-on-SiO_2 (blue data points) and corrugated graphene samples (red data points). Solid lines, Gaussian fits to data (two maxima were used to fit graphene-on-SiO_2 data).

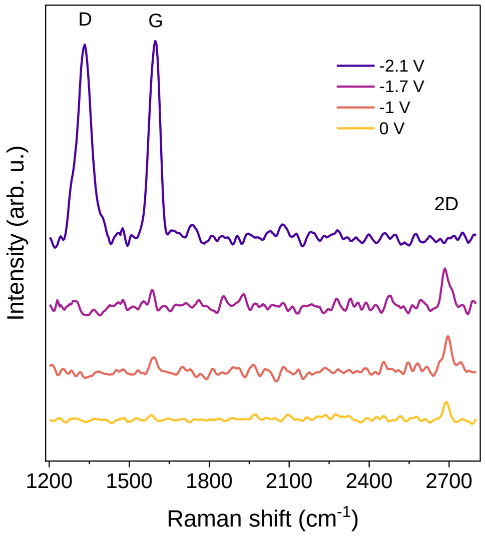

Supplementary Figure 10 | Raman characterisation of hydrogenation transition in corrugated graphene samples. Raman spectra of a corrugated graphene sample during a hydrogenation cycle. The signatures of the deuteration transition appear for a gate voltage of about -2.1 V vs NP, in agreement with the electronic characterisation.

Supplementary Table 1 | Statistics. Statistics for hydrogenation potential, maximum proton reduction current, maximum electronic current and number of samples for graphene-on-SiO_2, corrugated graphene and graphene-on-hBN.

Device type	Hydrogenation potential vs NP	Maximum proton reduction current (normalised by active area)	Maximum electronic current (normalised by flake width)	Number of samples
Graphene-on-SiO_2	-1.75 ± 0.08 V	0.19 ± 0.05 mA cm^{-2}	49 ± 10.4 mA m^{-1}	8
Corrugated graphene	-2.08 ± 0.13 V	4.3 ± 0.94 mA cm^{-2}	41 ± 12.3 mA m^{-1}	4
Graphene-on-hBN	-1.99 ± 0.02 V	0.23 ± 0.02 mA cm^{-2}	45 ± 7.4 mA m^{-1}	4
