# Supplementary material for: Mechanism of the electrochemical hydrogenation of graphene
Source: Nat Commun. 2025 Nov 28;16:10741. doi: 10.1038/s41467-025-65771-3 (PMC12663420; doi:10.1038/s41467-025-65771-3)
Supplement: Supplementary file 2 — Description of Additional Supplementary Files [file 41467_2025_65771_MOESM2_ESM.pdf]

Description of Additional Supplementary Files

File Name: Supplementary Data 1
Description: Raw Raman spectroscopy data for left inset of Figure 1a.

File Name: Supplementary Data 2
Description: Raw Raman spectroscopy data for top inset of Figure 2.

File Name: Supplementary Data 3
Description: Raw Raman spectroscopy data for Figure 3b.

File Name: Supplementary Data 4
Description: Raw Raman spectroscopy data for Supplementary Figure 8a and 8b.

File Name: Supplementary Data 5
Description: Raw Raman spectroscopy data for Supplementary Figure 10.
